# Japanese orthopaedic association back pain evaluation questionnaire (JOABPEQ) as an outcome measure for patients with low back pain: reference values in healthy volunteers

**DOI:** 10.1007/s00776-014-0693-1

**Published:** 2015-02-18

**Authors:** Hiroshi Hashizume, Shin-ichi Konno, Katsushi Takeshita, Mitsuru Fukui, Kazuhisa Takahashi, Kazuhiro Chiba, Masabumi Miyamoto, Morio Matsumoto, Yuichi Kasai, Masahiko Kanamori, Shunji Matsunaga, Noboru Hosono, Tsukasa Kanchiku, Hiroshi Taneichi, Nobuhiro Tanaka, Masahiro Kanayama, Takachika Shimizu, Mamoru Kawakami

**Affiliations:** 1 Department of Orthopaedic Surgery, Wakayama Medical University, 811-1 Kimiidera, Wakayama, 641-8510 Japan; 2 Department of Orthopaedic Surgery, Fukushima Medical University School of Medicine, Fukushima, Japan; 3 Department of Orthopaedic Surgery, The University of Tokyo, Tokyo, Japan; 4 Laboratory of Statistics, Osaka City University Faculty of Medicine, Osaka, Japan; 5 Department of Orthopedic Surgery, Graduate School of Medicine, Chiba University, Chiba, Japan; 6 Department of Orthopaedic Surgery, Kitasato University Kitasato Institute Hospital, Tokyo, Japan; 7 Department of Orthopedic Surgery, Nippon Medical School, Tama Nagayama Hospital, Tokyo, Japan; 8 Department of Orthopaedic Surgery, Keio University, Tokyo, Japan; 9 Department of Spinal Surgery and Medical Engineering, Mie University Graduate School of Medicine, Mie, Japan; 10 Department of Human Science 1, Faculty of Medicine, University of Toyama, Toyama, Japan; 11 Department of Orthopaedic Surgery, Imakiire General Hospital, Kagoshima, Japan; 12 Department of Orthopaedic Surgery, Osaka Kosei-nenkin Hospital, Osaka, Japan; 13 Department of Orthopaedic Surgery, Yamaguchi University Graduate School of Medicine, Yamaguchi, Japan; 14 Department of Orthopaedic Surgery, Dokkyo Medical University School of Medicine, Tochigi, Japan; 15 Department of Orthopaedic Surgery, Institute of Biomedical and Health Sciences, Hiroshima University, Hiroshima, Japan; 16 The Spine Center, Hakodate Central General Hospital, Hokkaido, Japan; 17 Department of Orthopaedic Surgery, Gunma Spine Center (Harunaso Hospital), Gunma, Japan; 18 Department of Orthopaedic Surgery, Wakayama Medical University Kihoku Hospital, Wakayama, Japan

## Abstract

**Background:**

In 2007, the Japanese orthopaedic association back pain evaluation questionnaire (JOABPEQ) was established to overcome the limitations of the original JOA scoring system developed in 1986. Although this new self-administered questionnaire is a more accurate outcome measure for evaluating patients with low back pain, physicians were unable to as certain the exact status of a patient at a single time point because of a lack of reference values. This study aimed to establish the reference values of JOABPEQ in different age and gender groups using data obtained from healthy volunteers.

**Methods:**

This study was conducted in 21 university hospitals and affiliated hospitals from October 2012 to July 2013. The JOABPEQ includes 25 questions that yield five domains to evaluate individuals with low back pain from five different perspectives. A total of 1,456 healthy volunteers (719 men, 737 women; age range, 20–89 years) answered the questionnaire. The differences in scores according to age and gender were examined by non-parametric tests.

**Results:**

The JOABPEQ scores significantly decreased with age in the domains of lumbar spine dysfunction, gait disturbance, and social life dysfunction. In these three domains, the median scores approached the 100 possible points in individuals aged 20–70 for both genders. However, the median scores for lumbar spine dysfunction and social life dysfunction decreased to 83.0 and 65.0–78.0 points, respectively, in individuals in their 80 s and 70–80 s, respectively; and the scores for gait disturbance decreased to 93.0 and 71.0 points for males and females in their 80 s. Overall, the median scores for pain-related and psychological disorders were 100 and 60.0–72.0 points, respectively.

**Conclusion:**

The reference values for JOABPEQ according to age and gender were established herein. Patients with low back pain should be evaluated with this new self-administered questionnaire taking these reference values into account.

## Introduction

In 1986, the Japan Orthopaedic Association (JOA) published the JOA scoring system, a specific instrument to measure the outcomes of patients with low back pain (LBP) [1]. Since then, this instrument has been widely used to evaluate the clinical results of various surgical and nonsurgical interventions for patients with LBP [2–4]. However, a major criticism of the JOA score is that it is not a patient-oriented measurement, but rather a physician-based one; and the patient’s perspective is now widely accepted to be essential for evaluating the results of interventions and making medical decisions [5]. The members of the subcommittee on evaluation for low back pain and cervical myelopathy, who also belong to the Clinical Outcomes Committee of the JOA, have composed a new self-administered questionnaire, the JOA Back Pain Evaluation Questionnaire (JOABPEQ), as a new outcome measure for patients with LBP [6–9] in order to solve the problems of the JOA score. The JOABPEQ provides specific, yet multidimensional, outcome measures for patients with LBP, including dysfunctions and disabilities caused by the disease, and psychosocial problems resulting from such dysfunctions and disabilities. The reliability and validity of the JOABPEQ have been verified by psychometric evaluations [7–9]. The scores for the JOABPEQ range from 0 to 100, with a higher score indicating a better health status. In clinical practice, physicians have been able to evaluate treatment efficacy by comparing patient scores before and after treatment, and to find better treatments by comparing the improvement rates among different treatment groups. However, physicians have been unable to ascertain the exact status of a patient at a single time point based simply on the scores, when reference values have not been established. Additionally, the influence of age and gender on the scores has not been fully examined, and concerns have arisen that an age-related decline in the scores may influence the overall evaluation. Therefore, reference values of physically unimpaired individuals in different age and gender groups are needed to further validate this new self-administered questionnaire. Accordingly, this study aimed to establish reference values of the JOABPEQ by gender in healthy volunteers in their 20 s up to 80 s.

## Participants and methods

**Fig. 1:**
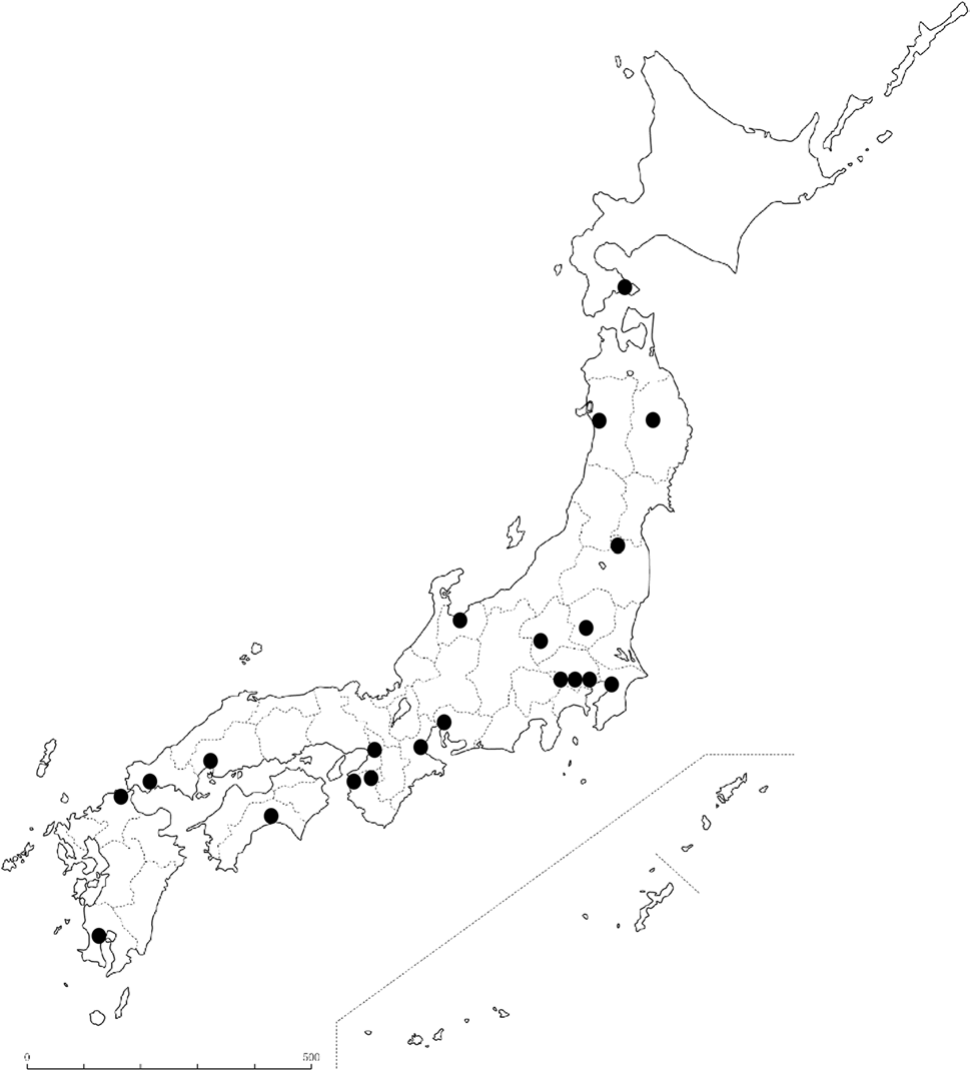
Distribution of the twenty-one institutions that participated in this study

This nationwide study was conducted in 21 university hospitals and their affiliated hospitals from October 2012 to July 2013 (Fig. 1). During this period, the 2 weeks at the end and beginning of the year were excluded from the survey since the holiday season may have influenced the participants psychosocially. At the planning of the study design, the authors, who comprised 17 board-certified spine surgeons and a medical statistician, all of whom were members of the Clinical Outcome Committee of the Japanese Society for Spine Surgery and Related Research, discussed and established the selection criteria of the participants. A summary of the selection criteria is shown in Table 1. The target population was healthy individuals who were self-supporting and required no medical treatments for orthopedic diseases. The target age range was 20–89 years, and the participants were divided into groups according to gender (male/female) and age (20, 30, 40, 50, 60, 70, and 80 s). We aimed to survey five healthy individuals within each age group and from both genders (i.e., 5 persons × 7 age groups × both genders = 70 persons in total) at each institution in order to achieve a sufficient statistical power. The participants were recruited at each hospital by invitation of the research collaborators, who are all board-certified orthopedic surgeons. Patients’ relatives, hospital employees, and persons concerned with the employees were candidates for the study as long as the person was not a medical professional (e.g., physician, nurse, therapist, etc.).Subjects were excluded if they were unable to understand the questionnaire because of cognitive impairment, were under treatment for orthopedic disorders at a medical institution, had a history of previous lumbar surgery. However, subjects with LBP were not excluded if they were judged to be able to perform daily their living activities unimpaired. Similarly, subjects receiving alternative medicine (e.g., acupuncture, massage, Judo therapy) were not excluded if the person was judged to be unimpaired. These two exceptions were made based on the following consensus after careful discussions by the authors: (1) since the prevalence of LBP is very high [10, 11], an appreciable number of subjects could potentially be prevented from participating if we excluded those who had mild LBP without an impact on activities of daily living, and as a result, the true status of the Japanese population might not be reflected; (2) Japanese individuals receiving alternative medicines usually do not have severe medical conditions, because they perceive alternative medicines as more casual and receive them very often; and (3) thus, subjects with mild LBP and/or those who are receiving alternative medicine could be included if the person was able to perform their usual activities of daily living unimpaired. The eligibility was confirmed by the board-certified orthopedic surgeons at each institute.

**Table 1:** Subject selection criteria

Inclusion criteria
Age between 20 and 89 years
Self-supporting and requires no medical treatment for orthopedic diseases^a^
Exclusion criteria
Cannot understand the questionnaire due to cognitive impairment
Under treatment for orthopedic disorders at a medical institution^a^
Previous operation of the lumbar spine
Medical professionals (e.g., physician, nurse, therapist, etc.)

^a^Subjects with low back pain, judged to be able to perform daily their living activities unimpaired, were not excluded, even if they were receiving alternative medicine

This study was conducted in accordance with the World Medical Association Declaration of Helsinki [12], and approved by the institutional review board of each institution. All subjects provided informed consent prior to enrollment in the study.

### Self-administered questionnaires

JOABPEQ includes 25 questions that yield five domains: pain-related disorders, lumbar spine dysfunction, gait disturbance, social life dysfunction, and psychological disorders. Visual analogue scales (VASs) were used to evaluate the degree of LBP and pain or numbness in the buttocks and lower limbs with respect to the relevant items in the JOABPEQ. The participants recalled their physical condition during the previous week and circled the number of the answer that best applied to their condition for each question. If the condition changed depending on the day or time, they were asked to select the answer representing the “worst” condition. The score of each domain was calculated according to the official guidelines and ranged from 0 to 100 points, which is proportional to the patient’s clinical condition [7–9].

**Fig. 2:**
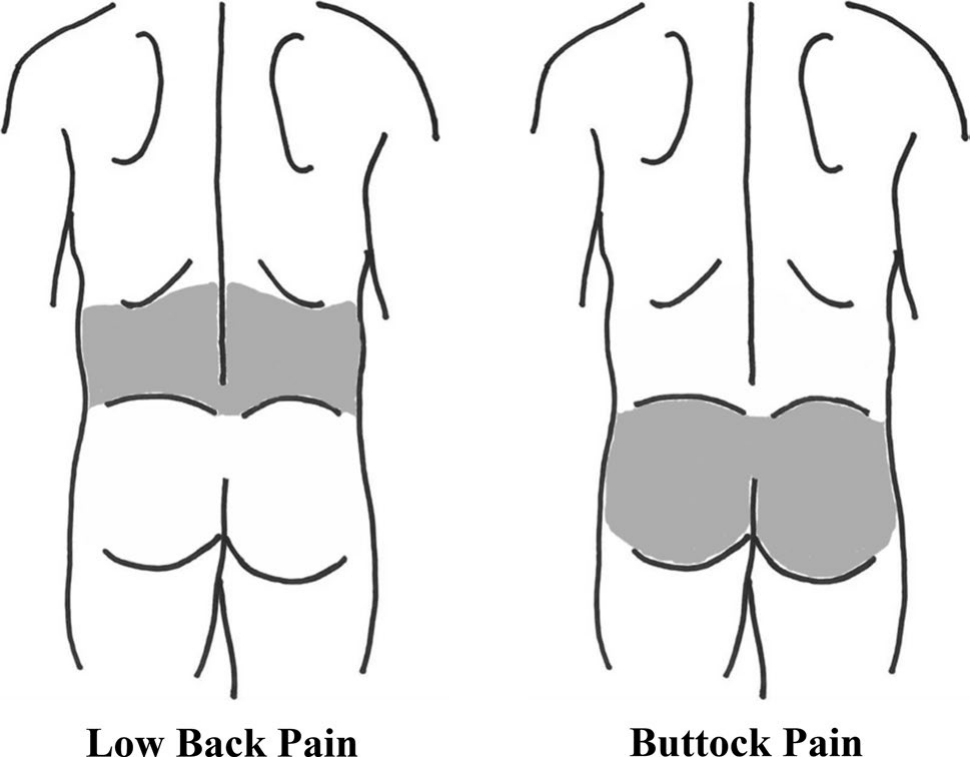
The schematic diagram explaining the areas of low back pain and buttock pain for the responders

In addition, the participants were asked to respond to a question concerning the presence of LBP during the past month to evaluate its prevalence. In general, LBP is defined as pain and discomfort localized between the lowest costal margin and the inferior gluteal folds [13]. In the present study, however, the prevalence of LBP was examined by two different definitions as follows: LBP-A, the pain and discomfort localized between the costal margin and the iliac crest (therefore excluding the buttocks); and LBP-B, the pain and discomfort localized between the costal margin and the inferior gluteal folds. The LBP-A was examined for the purpose of maintaining consistency with the concepts of the JOABPEQ, in which VAS scores are used to separately evaluate the intensity of LBP and buttock pain; andthe LBP-B was examined in order to determine the prevalence of LBP, thus allowing the results to be more readily compared to those of previous studies. To obtain information of both types of LBP, the participants were asked whether they had experienced LBP localized between the costal margin and the iliac crest and/or buttock pain localized between the iliac crest and the inferior gluteal folds that continued for more than 24 h during the past month. A schematic diagram explaining the definitions of LBP-A and buttock pain was provided with the questionnaire to the responders (Fig. 2). Moreover, the responders were provided the additional information that pain in the lower limbs did not include knee joint pain.

### Statistical analysis

The functional score was calculated only if all questions for that particular domain were answered. Domains in which the participant did not answer all of the questions or provided inappropriate answers due to failure to follow instructions were excluded from the analysis. In the descriptive statistics, quartiles including median values were used for depicting the distribution of the scores of each domain in JOABPEQ, stratified by gender and age (i.e., 20–80 s), as the five functional scores have not been confirmed to follow a normal distribution [9]. Similarly, the VAS scores were also described in a non-parametric manner because the normality of score distribution in the current study was denied by the Shapiro–Wilk test. The Steel–Dwass test was used for multiple comparisons among different generations, and the Jonckheere–Terpstra test was used to identify trends with regard to age for each domain. A *p* value <0.05 was considered significant.

## Results

A total of 1,469 healthy volunteers who were self-supporting and received no medical treatments for orthopedic diseases answered the survey. Of these, 9 individuals were excluded because of age-related criteria (i.e., they were <20 or >90 years old), and 4 individuals were excluded because of a lack of gender information. Thus, the answers of 1,456 volunteers (719 males and 737 females) were used for the analysis. The number of subjects and prevalence of LBP in the different age groups according to gender are provided in Table 2. The prevalence of LBP-A and LBP-B were 10.5 % (11.8 % in males, 9.2 % in females) and 11.5 % (12.6 % in males, 10.4 % in females), respectively.

**Table 2:** Distribution of age, gender, and prevalence of low back pain in the 1,456 volunteers

Age groups (years)	Male			Female		
	*n*	LBP-A	LBP-B	*n*	LBP-A	LBP-B
20–29	106	16 (15.1 %)	17 (16.0 %)	126	9 (7.1 %)	9 (7.1 %)
30–39	107	8 (7.5 %)	9 (8.4 %)	104	3 (2.9 %)	3 (2.9 %)
40–49	109	11 (10.1 %)	11 (10.1 %)	110	5 (4.5 %)	5 (4.5 %)
50–59	109	12 (11.0 %)	12 (11.0 %)	105	11 (10.5 %)	14 (13.3 %)
60–69	99	12 (12.1 %)	12 (12.1 %)	98	10 (10.2 %)	11 (11.2 %)
70–79	97	15 (15.5 %)	18 (18.6 %)	100	15 (15.0 %)	17 (17.0 %)
80–89	92	11 (12.0 %)	12 (13.0 %)	94	15 (16.0 %)	18 (19.1 %)
Total	719	85 (11.8 %)	91 (12.6 %)	737	68 (9.2 %)	77 (10.4 %)

Data are presented as *n* (%)

*LBP-A* low back pain defined as pain and discomfort localized between the costal margin and the iliac crest, *LBP-B* low back pain defined as pain and discomfort localized between the costal margin and the inferior gluteal folds

### Pain-related disorders

**Fig. 3:**
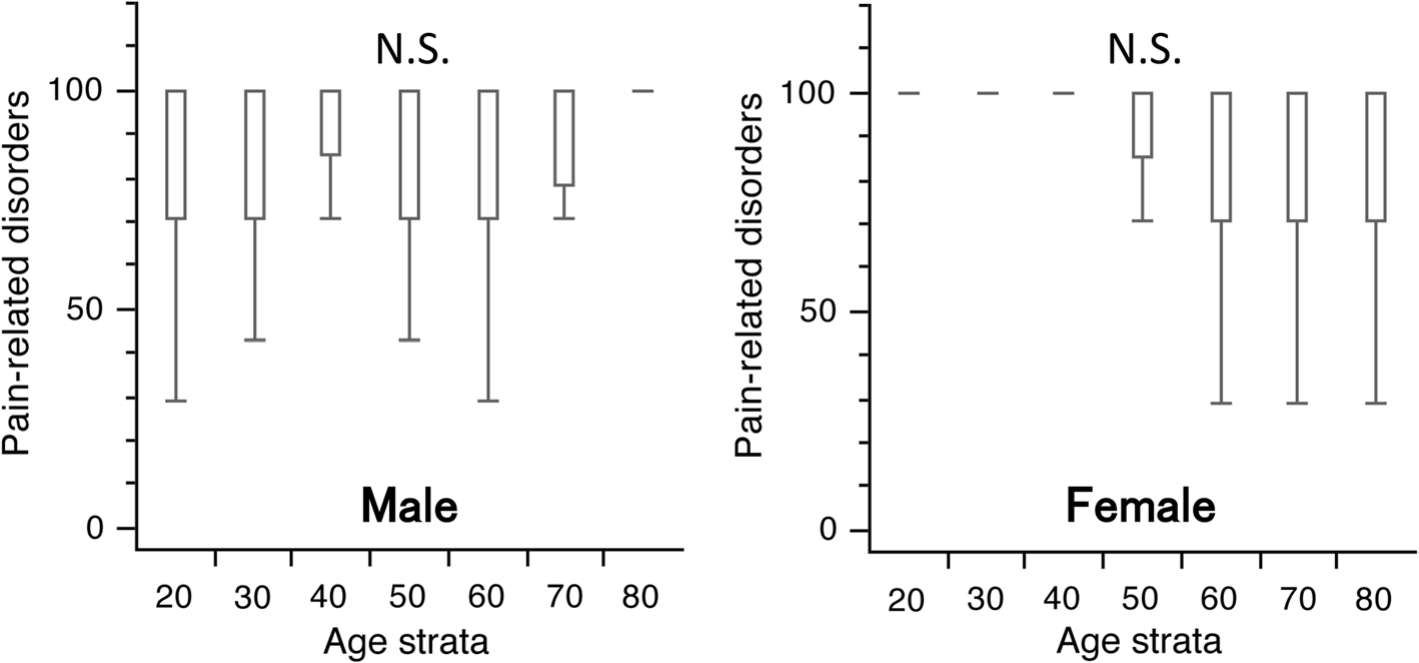
*Box* and *whisker* plots of scores for pain-related disorders in the Japanese Orthopaedic Association Back Pain Evaluation Questionnaire by gender and age group. The *lines* on the *box* correspond to the quartiles. Whiskers indicate the furthest point within 1.5× interquartile range from the box. There were no significant differences in the scores between any age groups in both genders (Steel–Dwass test). The scores had a tendency to increase with age in males and to decrease with age in females (Jonckheere–Terpstra test; *p* < 0.001 for both). N.S., not significant

Overall, for both genders, the median score was 100. Among males, the first quartiles ranged between 71.0 and 85.5 in individuals in their 20–70 s, while it was 100 in subjects in their 80 s. Among females, the first quartiles ranged between 71.0 and 85.5 in individuals in their 50–80 s, while it was 100 in individuals in their 20–40 s. For both genders, there were no significant differences in the scores between any age group (Fig. 3).

### Lumbar spine dysfunction

**Fig. 4:**
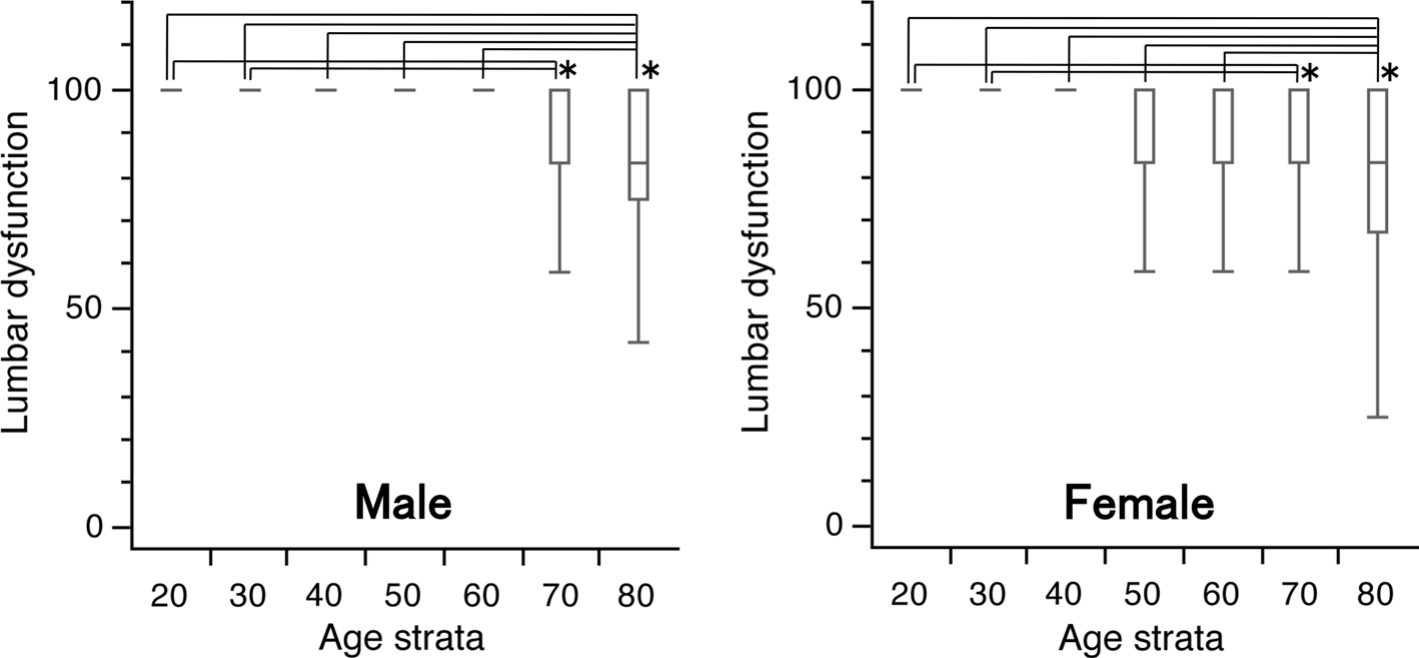
*Box* and *whisker* plots of scores for lumbar spine dysfunction in the Japanese Orthopaedic Association Back Pain Evaluation Questionnaire by gender and age group. The *lines* on the *box* correspond to the quartiles. Whiskers indicate the furthest point within 1.5× interquartile range from the box. Significant differences were observed in the scores between the younger generation in their 20 and 30 s and subjects in their 70 s, and between subjects in their 20–60 s and those in their 80 s in both genders (Steel–Dwass test; *p < 0.05). The scores had a tendency to decrease with age in both genders (Jonckheere–Terpstra test; *p* < 0.001)

For both genders, the median scores were 100 in subjects in their 20–70 s, while it was 83.0 in subjects in their 80 s. Among males, the first quartiles were all 100 in individuals in their 20–60 s, but decreased to 83.0 and 75.0 for those in their 70 and 80 s, respectively. Among females, the first quartiles were 100 in subjects in their 20–40 s, but this decreased to 83.0 in women in their 50–70 s, and further decreased to 67.0 points among those in their 80 s. Significant differences were observed in the scores between the younger subjects in their 20 s and 30 s compared to subjects in their 70 s(*p* < 0.05), and between individuals in their 20 s to 60 s compared to those in their 80 s (*p* < 0.05) in both genders (Fig. 4).

### Gait disturbance

**Fig. 5:**
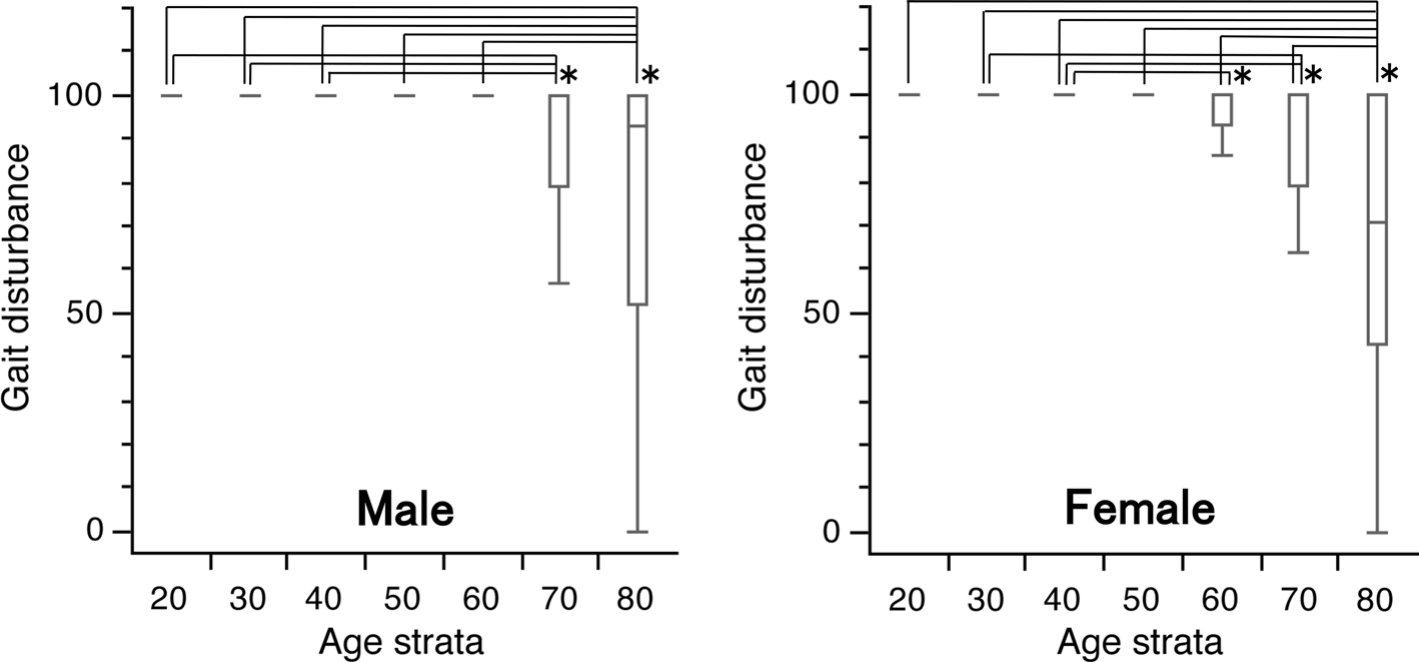
*Box* and *whisker* plots of scores for gait disturbance in the Japanese Orthopaedic Association Back Pain Evaluation Questionnaire by gender and age group. The *lines* on the *box* correspond to the quartiles. Whiskers indicate the furthest point within 1.5× interquartile range from the box. Significant differences were observed in the scores between younger subjects in their 20–60 s and elderly subjects in their 80 s for both genders, between male subjects in their 20–40 s vs. in their 70 s, between female subjects in their 40–60 s, in their 30 s and 40 vs. 70 s, and in their 70 s vs. 80 s (Steel–Dwass test; *p < 0.05 for all). The scores had a tendency to decrease with age in both genders (Jonckheere–Terpstra test; *p* < 0.001)

For males, the median scores were all 100 for subjects in their 20–70 s, but this value decreased to 93.0 points for those in their 80 s. The first quartiles were 100 for subjects in their 20–60 s, but decreased to 79.0 and 51.8 for those in their 70 and 80 s, respectively. Significant differences were seen between male subjects in their 20–40 s and those in their 70 s, and between male individuals in their 20–60 s and those in their 80 s. For females, the median scores were 100 for subjects in their 20–70 s and 71.0 in women in their 80 s. The first quartiles were 100 in their 20–50 s, but this decreased to 93.0, 79.0, and 43.0 in their 60, 70, and 80 s, respectively. Significant differences were observed in the scores between female subjects in their 40 vs. 60 s, in their 30 and 40 s vs. 70 s, and in their 70 vs. 80 s (*p* < 0.05 for all; Fig. 5).

### Social life dysfunction

**Fig. 6:**
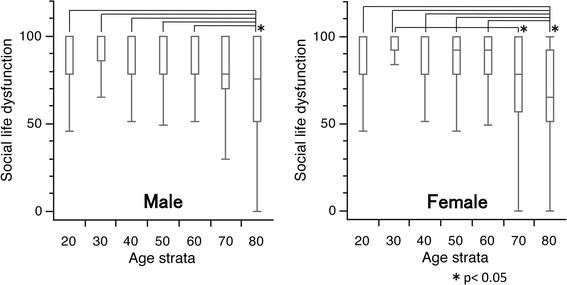
*Box* and *whisker* plots of scores for social life function in the Japanese Orthopaedic Association Back Pain Evaluation Questionnaire by gender and age group. The *lines* on the *box* correspond to the quartiles. *Whiskers* indicate the furthest point within 1.5× interquartile range from the *box*. Significant differences were observed in the scores between subjects in their 20–60 s and those in their 80 s (Steel–Dwass test; *p < 0.05) in both genders. Moreover, a significant difference in the scores between female subjects in their 30 and 70 s was observed (Steel–Dwass test; *p < 0.05). The scores had a tendency to decrease with age in both genders (Jonckheere–Terpstra test; *p* < 0.001)

For males, the median scores were 100 for subjects in their 20–60 s, but decreased to 78.0 and 75.5 for those in their 70 and 80 s, respectively. The first quartile was highest for subjects in their 30 s, with a score of 86.0 points. The corresponding scores were 78.0 for subjects in their 20, 40, 50, and 60 s; and 70.0 and 51.0 in subjects in their 70 and 80 s, respectively. For females, the median scores were 100 for subjects in their 20–40 s, 92 for those in their 50 and 60 s, and 78.0 and 65.0 in subjects in their 70 and 80 s, respectively. The first quartile was the highest for subjects in their 30 s, with a score of 92.0 points. The corresponding values were 78.0 for subjects in their 20, 40, 50, and 60 s; but this decreased to 57.0 and 51.0 in individuals in their 70 and 80 s, respectively. Significant differences were observed in the scores between the subjects in their 20–60 s compared to those in their 80 s (*p* < 0.05) in both genders, and between females in their 30 vs. 70 s (*p* < 0.05) (Fig. 6).

### Psychological disorders

**Fig. 7:**
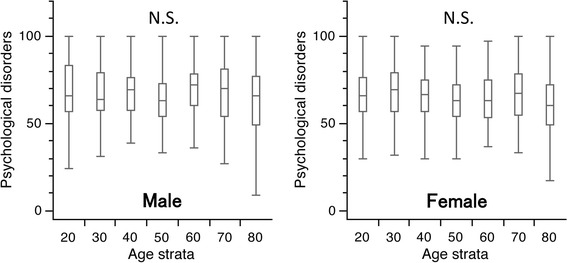
*Box* and *whisker* plots of scores for psychological disorders in the Japanese Orthopaedic Association Back Pain Evaluation Questionnaire by gender and age group. The lines on the box correspond to the quartiles. *Whiskers* indicate the furthest point within 1.5× interquartile range from the *box*. There were no significant differences in the scores between any age groups in both genders (Steel–Dwass test). The scores had a tendency to decrease with age in both genders (Jonckheere–Terpstra test, *p* < 0.001)

For both genders, the median and the first quartile scores ranged between 60.0–72.0 and 49.0–60.0 across all generations, respectively. Moreover, the third quartile scores were 72.0–83.5 points across all generations in both genders. There were no significant differences in the scores between any age group in both genders (Fig. 7).

### Age trends among the various functional domains

Based on the results of the Jonckheere–Terpstra test, the scores tended to decrease with age in all domains in both genders, with the exception of the score for pain-related disorders, which showed a tendency to increase among males.

### VASs

The median scores for all VAS score ranged between 0.0 and 5.0 mm, with the majority being 0.0 mm in all age groups for both genders. For males, the third quartile values in the different age groups for LBP-A, pain in the buttocks and lower limbs, and numbness in the buttocks and lower limbs ranged between 17.0–27.0, 0.0–14.0, and 0.0–8.0, respectively. For females, the three VAS domains ranged between 10.0–30.5, 0.0–29.0, and 0.0–13.0, respectively.

There were no significant differences in the VAS scores for LBP-A among different age groups in both genders, or for pain or numbness of the buttocks and lower limbs among different age groups in males. However, in females, significant differences in the VAS scores for pain in the buttocks and lower limbs were seen between subjects in their 20–40 s and those in their 80 s. Further, a significant difference was seen in the VAS scores for numbness in the buttocks and lower limbs between female participants in their 20–30 s and those in their 80 s. The VAS scores of all regions tended to increase with age in both genders, with the exception of the VAS score for LBP in males, which did not change with age.

As described above, the overall prevalence of LBP-A was 10.5 %. The quartile value (median [first quartile–third quartile]) of the VAS score for LBP-A in the participants who responded “Yes” to the question asking the existence of LBP-A in the past month was 39 (20.5–58).

## Discussion

The purpose of this study was to provide reference values of the JOABPEQ in healthy adult populations who do not require medical assistance. We have established the reference values of JOABPEQ in the Japanese population for the first time, based on the data obtained from 1,456 healthy volunteers recruited nationwide at 21 centers. The reference values should provide physicians with useful information on the status of a specific patient by comparing his or her scores with the reference values.

The JOABPEQ was designed to evaluate the overall health status (i.e., both physical and psychosocial disorders) in patients with LBP, and may be suitable for following changes in the status of each patient; however, it may unsuitable to directly compare such changes among different patients [9]. Currently, a treatment is judged as “effective” for a particular patient if: (1) the patient provided answers to all the questions necessary to calculate a domain score and showed an increase of ≥20 points after the treatment, or (2) the functional score after treatment exceeds 90 points even if the answer for an unanswered question is assumed as the worst possible choice [9]. Our findings suggest that these values may require adjustment by age and gender. Moreover, the values obtained here for the various age groups in both genders will serve as the reference when comparing scores of an individual or group to those of other individuals or groups.

The prevalence of degenerative diseases of the lumbar spine, including disc degeneration [14] and lumbar spinal stenosis [15, 16], was reported to increase with age in population-based cohort studies in Japan. Similarly, the prevalence of osteoporosis was found to increase with age in the Japanese population and to lead to a surge in the incidence of vertebral fractures in older subjects, especially in women, in other studies [17, 18]. Based on these reports, we assumed that physical function related to the lumbar spine would decline with age. The differences in the JOABPEQ scores according to age, sex, and disease type have been previously reported based on patient data [19]. Our findings are in conformity with these expectations, and indicate a need to establish reference values for newly developed questionnaires.

In the five domains of JOABPEQ, the scores for lumbar spine dysfunction, gait disturbance, and social life dysfunction decreased significantly with age, while the influence of aging was small for pain-related disorders and psychological disorders. Of particular note, the median scores for pain-related disorders were 100 points for all age groups in both genders. Meanwhile, the median scores for psychological disorders were 60–70 points for all age groups in both genders, with the exception of males in their 60 s (median score: 72.0 points). Additionally, third quartile scores of >90 points were not observed in any age group for either gender in the domain of psychological disorders. Hence, a reconsideration is needed for judging the effectiveness of a treatment based on a score of >90 points for this domain.

Our study revealed that there are variations and different trends in the reference values of scores for five domains of JOABPEQ in different age groups in both genders. This result is not surprising, as the JOABPEQ is partly derived from the Roland Morris Questionnaire and the MOS 36-Item Short-Form Health Survey (SF-36) [6]. The SF-36 is the most widely used survey to measure the health-related quality of life, and the reference values for various parameters (e.g., general health perceptions, social functioning) have been shown to vary considerably in Japanese adults [20]. Accordingly, the development of a norm-based scoring system similar to SF-36 [20] may be needed to further understand the JOABPEQ scores.

Although VAS for LBP and pain or numbness in the buttocks and lower limbs are relevant items in the JOABPEQ, we did not statistically analyze the relationship between VAS values and JOABPEQ scores. The main reason for this was the purpose of this study, which was to establish the reference values of the JOABPEQ according to gender in healthy volunteers of different ages. Instead, we only presented the representative values of the VASs to provide a better understanding of the study population. Most of the VAS domains were significantly influenced by age. However, the median VAS values for LBP-A and pain or numbness in the buttocks and lower limbs were generally <10 mm, with most being 0 mm in all age groups for both genders. The third quartile VAS values for the three regions ranged between 0.0 and 30.5 across all generation in both genders. Collins et al. reported that 30–54 mm on the VAS was considered as moderate pain based on the distribution of VAS scores corresponding to a 4-point categorical scale (none, mild, moderate, and severe) [21]. Therefore, most of the participants in the current study did not have severe low back pain, which is consistent with the inclusion criteria of our target population. Nevertheless, there is a possibility that subjects with LBP causing impairment might have been included in the study. However, we believe that reference values and statistical results provided here remain robust, because outliers do not easily affect the quartiles and the results of non-parametric tests. In the present study, 11.5 % of the subjects fulfilled the definition for LBP-B, which is less than the 25.2 % prevalence rate estimated from 20,044 respondents of a recent Internet survey of the general Japanese population using the same definition [10]. The prevalence of LBP and the distribution of VAS scores would support the validity of our target population.

This study has several limitations. First, anthropometric data, including body mass index, exercise habits, the detailed medical history, and general health (e.g., mental status) of the volunteers were not fully assessed, and the age-related degenerative changes of the lumbar spine were not investigated. Physical and mental conditions, therefore, may have affected the scores. Second, the data sampling was not randomized from the national resident record, and therefore the reference values acquired in this study are not population-based. Third, the physicians, who were aware of the selection criteria of this study, conducted the recruitment of the participants. A selection bias may occur if the physicians had any expectations about the results. However, for this point, we believe that the pre-screening bias was minimized because this study was not a trial but rather a cross-sectional observational study. Fourth, the agreement rate for the study participation among the candidates was not fully recorded, although we believe that it was almost 100 %, since the subjects were pre-screened by the physicians. The selection criteria for “healthy volunteers” and potential biases described above should be taken into account when using the values obtained in this study as reference values.

In conclusion, in this study, reference values of JOABPEQ were established for healthy volunteers. Physicians should be aware that the JOABPEQ scores may vary by domain, gender, and age. The reference values derived herein from stratified sampling will help improve the understanding of the JOABPEQ scores, and may help identify the most appropriate treatments or medical services for patients with LBP.

## Appendix

See also Tables 3, 4, 5, 6, 7, 8, 9, 10.

**Table 3:** Distribution of scores for domain of “Pain related disorders”

		Male							Female							
Age		20–29	30–39	40–49	50–59	60–69	70–79	80–89		20–29	30–39	40–49	50–59	60–69	70–79	80–89
Number	Valid	105	106	109	109	98	96	91	Valid	125	104	109	105	97	97	93
	Invalid	1	1	0	0	1	1	1	Invalid	1	0	1	0	1	3	1
Average		85.1	87.8	88.7	88.7	84.9	87.0	87.7		91.5	93.2	91.2	88.2	87.4	85.4	82.0
95% CI		80.6–89.6	83.4–92.2	84.3–93.0	84.8–92.5	80.2–89.6	82.1–92.0	82.3–93.1		88.4–94.6	90.0–96.5	87.9–94.5	83.8–92.7	82.7–92.2	80.2–90.5	76.2–87.8
Percentile	10.0	43.0	62.6	43.0	71.0	43.0	43.0	43.0	10.0	71.0	71.0	71.0	43.0	43.0	43.0	43.0
	25.0	71.0	71.0	85.5	71.0	71.0	78.3	100.0	25.0	100.0	100.0	100.0	85.5	71.0	71.0	71.0
	50.0	100.0	100.0	100.0	100.0	100.0	100.0	100.0	50.0	100.0	100.0	100.0	100.0	100.0	100.0	100.0
	75.0	100.0	100.0	100.0	100.0	100.0	100.0	100.0	75.0	100.0	100.0	100.0	100.0	100.0	100.0	100.0
	90.0	100.0	100.0	100.0	100.0	100.0	100.0	100.0	90.0	100.0	100.0	100.0	100.0	100.0	100.0	100.0

*CI* confidence interval

**Table 4:** Distribution of scores for domain of “Lumbar spine dysfunction”

		Male							Female							
Age		20–29	30–39	40–49	50–59	60–69	70–79	80–89		20–29	30–39	40–49	50–59	60–69	70–79	80–89
Number	Valid	106	107	109	108	97	95	90	Valid	124	104	109	103	95	99	90
	Invalid	0	0	0	1	2	2	2	Invalid	2	0	1	2	3	1	4
Average		96.1	95.1	93.3	92.0	92.4	85.7	80.1		96.9	97.9	95.0	90.2	89.9	85.9	75.1
95% CI		94.2–98.1	92.3–97.8	90.4–96.2	88.4–95.6	88.9–95.9	81.4–90.0	74.9–85.3		95.3–98.5	96.6–99.2	92.8–97.2	86.3–94.0	86.0–93.8	81.4–90.5	69.5–80.7
Percentile	10.0	83.0	83.0	83.0	73.3	65.2	50.0	33.9	10.0	83.0	92.0	83.0	58.0	58.0	50.0	25.8
	25.0	100.0	100.0	100.0	100.0	100.0	83.0	75.0	25.0	100.0	100.0	100.0	83.0	83.0	83.0	67.0
	50.0	100.0	100.0	100.0	100.0	100.0	100.0	83.0	50.0	100.0	100.0	100.0	100.0	100.0	100.0	83.0
	75.0	100.0	100.0	100.0	100.0	100.0	100.0	100.0	75.0	100.0	100.0	100.0	100.0	100.0	100.0	100.0
	90.0	100.0	100.0	100.0	100.0	100.0	100.0	100.0	90.0	100.0	100.0	100.0	100.0	100.0	100.0	100.0

*CI* confidence interval

**Table 5:** Distribution of scores for domain of “Gait disturbance”

		Male							Female							
Age		20–29	30–39	40–49	50–59	60–69	70–79	80–89		20–29	30–39	40–49	50–59	60–69	70–79	80–89
Number	valid	106	105	109	108	97	96	88	Valid	126	104	109	105	96	95	92
	invalid	0	2	0	1	2	1	4	Invalid	0	0	1	0	2	5	2
Average		99.2	98.1	96.6	95.3	95.2	87.2	74.3		97.0	98.0	98.0	93.5	92.5	88.0	69.5
95% CI		98.5–99.9	96.3–99.9	94.5–98.7	93.0–97.5	92.6–97.7	82.9–91.5	67.7–80.8		95.3–98.7	96.4–99.6	96.5–99.6	90.2–96.8	89.2–95.8	83.6–92.4	63.4–75.7
Percentile	10.0	100.0	100.0	93.0	79.0	84.6	43.0	28.2	10.0	93.0	96.5	100.0	71.0	71.0	64.0	23.4
	25.0	100.0	100.0	100.0	100.0	100.0	79.0	51.8	25.0	100.0	100.0	100.0	100.0	93.0	79.0	43.0
	50.0	100.0	100.0	100.0	100.0	100.0	100.0	93.0	50.0	100.0	100.0	100.0	100.0	100.0	100.0	71.0
	75.0	100.0	100.0	100.0	100.0	100.0	100.0	100.0	75.0	100.0	100.0	100.0	100.0	100.0	100.0	100.0
	90.0	100.0	100.0	100.0	100.0	100.0	100.0	100.0	90.0	100.0	100.0	100.0	100.0	100.0	100.0	100.0

*CI* confidence interval

**Table 6:** Distribution of scores for domain of “Social life dysfunction”

		Male							Female							
Age		20–29	30–39	40–49	50–59	60–69	70–79	80–89		20–29	30–39	40–49	50–59	60–69	70–79	80–89
Number	valid	105	106	107	107	98	93	88	Valid	125	102	108	103	91	93	89
	invalid	1	1	2	2	1	4	4	Invalid	1	2	2	2	7	7	5
Average		88.8	90.2	87.7	87.5	87.7	79.5	68.9		87.5	92.7	88.9	86.0	83.9	78.8	67.7
95% CI		85.6–92.1	87.1–93.3	84.5–91.0	84.0–91.0	84.4–91.0	74.9–84.1	62.8–74.9		84.5–90.4	90.2–95.3	85.9–92.0	82.6–89.5	80.1–87.7	74.1–83.5	62.5–73.0
Percentile	10.0	57.0	65.0	57.0	57.0	57.0	51.0	24.0	10.0	57.0	78.0	58.8	57.0	51.0	51.0	30.0
	25.0	78.0	86.0	78.0	78.0	78.0	70.0	51.0	25.0	78.0	92.0	78.0	78.0	78.0	57.0	51.0
	50.0	100.0	100.0	100.0	100.0	100.0	78.0	75.5	50.0	100.0	100.0	100.0	92.0	92.0	78.0	65.0
	75.0	100.0	100.0	100.0	100.0	100.0	100.0	100.0	75.0	100.0	100.0	100.0	100.0	100.0	100.0	92.0
	90.0	100.0	100.0	100.0	100.0	100.0	100.0	100.0	90.0	100.0	100.0	100.0	100.0	100.0	100.0	100.0

*CI* confidence interval

**Table 7:** Distribution of scores for domain of “Psychological disorders”

		Male							Female							
Age		20–29	30–39	40–49	50–59	60–69	70–79	80–89		20–29	30–39	40–49	50–59	60–69	70–79	80–89
Number	Valid	104	105	108	108	97	95	89	Valid	125	103	108	104	92	97	92
	Invalid	2	2	1	1	2	2	3	Invalid	1	1	2	1	6	3	2
Average		68.5	66.4	67.9	64.3	69.2	68.0	62.2		66.9	69.1	65.7	62.8	64.5	67.1	59.4
95% CI		65.1–71.9	63.5–69.3	65.2–70.5	61.5–67.1	66.3–72.1	64.8–71.2	58.4–66.1		64.2–69.5	66.2–72.0	63.2–68.2	60.0–65.6	61.7–67.4	63.7–70.6	55.4–63.5
Percentile	10.0	49.0	49.0	49.9	45.9	50.4	46.8	36.0	10.0	51.0	49.0	49.0	45.5	49.0	47.4	39.0
	25.0	57.0	57.5	57.8	54.0	60.0	54.0	49.5	25.0	57.0	57.0	57.0	54.0	53.3	55.0	49.0
	50.0	66.0	64.0	69.0	63.0	72.0	70.0	66.0	50.0	66.0	69.0	66.5	63.0	63.0	67.0	60.0
	75.0	83.5	79.0	76.0	73.0	78.0	81.0	77.0	75.0	76.0	79.0	75.0	72.0	75.0	78.0	72.0
	90.0	94.0	84.0	87.0	87.0	86.2	87.0	83.0	90.0	85.8	87.0	82.0	81.5	83.7	91.2	83.7

*CI* confidence interval

**Table 8:** Distribution of scores in VAS for low back pain

		Male							Female							
Age		20–29	30–39	40–49	50–59	60–69	70–79	80–89		20–29	30–39	40–49	50–59	60–69	70–79	80–89
Number	Valid	103	106	107	103	90	87	77	Valid	122	103	106	103	91	90	84
	Invalid	3	1	2	6	9	10	15	Invalid	4	1	4	2	7	10	10
Average		11.5	13.0	10.6	11.5	12.1	15.2	14.1		9.8	8.1	10.3	12.0	11.4	14.6	18.1
95% CI		8.2–14.9	9.3–16.6	7.4–13.8	8.1–14.9	8.0–16.2	10.4–20.1	8.9–19.2		6.8–12.7	5.0–11.2	7.2–13.3	8.4–15.5	7.8–15.0	10.2–19.1	13.0–23.3
Percentile	10.0	0.0	0.0	0.0	0.0	0.0	0.0	0.0	10.0	0.0	0.0	0.0	0.0	0.0	0.0	0.0
	25.0	0.0	0.0	0.0	0.0	0.0	0.0	0.0	25.0	0.0	0.0	0.0	0.0	0.0	0.0	0.0
	50.0	1.0	0.0	0.0	0.0	0.0	3.0	0.0	50.0	0.0	0.0	0.0	3.0	0.0	0.0	5.0
	75.0	20.0	20.3	18.0	17.0	19.3	27.0	17.0	75.0	13.3	10.0	16.3	16.0	19.0	23.0	30.5
	90.0	33.8	41.0	30.2	35.2	46.4	51.4	52.4	90.0	33.4	30.0	33.3	43.4	39.8	54.0	55.0

*CI* confidence interval

**Table 9:** Distribution of scores in VAS for pain in buttock(s) and lower limb(s)

		Male							Female							
Age		20–29	30–39	40–49	50–59	60–69	70–79	80–89		20–29	30–39	40–49	50–59	60–69	70–79	80–89
Number	Valid	104	106	107	104	92	86	78	Valid	123	103	107	103	91	88	84
	Invalid	2	1	2	5	7	11	14	Invalid	3	1	3	2	7	12	10
Average		3.5	3.9	3.5	3.6	5.9	11.0	10.0		3.2	3.0	4.8	8.5	7.8	9.5	17.3
95% CI		1.8–5.2	2.0–5.8	1.9–5.1	2.0–5.1	3.0–8.7	6.3–15.6	5.9–14.1		1.5–5.0	1.3–4.8	2.4–7.2	4.9–12.1	4.8–10.8	5.5–13.4	11.9–22.7
Percentile	10.0	0.0	0.0	0.0	0.0	0.0	0.0	0.0	10.0	0.0	0.0	0.0	0.0	0.0	0.0	0.0
	25.0	0.0	0.0	0.0	0.0	0.0	0.0	0.0	25.0	0.0	0.0	0.0	0.0	0.0	0.0	0.0
	50.0	0.0	0.0	0.0	0.0	0.0	0.0	0.0	50.0	0.0	0.0	0.0	0.0	0.0	0.0	1.5
	75.0	0.0	0.0	0.0	1.0	2.8	10.5	14.0	75.0	0.0	0.0	0.0	9.0	13.0	11.0	29.0
	90.0	13.5	16.8	16.4	12.0	23.8	47.6	38.7	90.0	11.0	14.8	18.8	30.6	27.8	35.7	59.0

*CI* confidence interval

**Table 10:** Distribution of scores in VAS for numbness in buttock(s) and lower limb(s)

		Male							Female							
Age		20–29	30–39	40–49	50–59	60–69	70–79	80–89		20–29	30–39	40–49	50–59	60–69	70–79	80–89
Number	Valid	105	106	108	106	93	83	79	Valid	123	101	107	102	90	86	83
	Invalid	1	1	1	3	6	14	13	Invalid	3	3	3	3	8	14	11
Average		2.4	2.3	2.6	3.0	4.1	9.1	8.7		1.5	0.7	3.1	4.7	4.8	4.8	11.8
95% CI		1.0–3.8	0.9–3.7	1.2–3.9	1.6–4.3	1.4–6.7	4.8–13.5	4.4–13.1		0.3–2.7	0.0–1.4	1.1–5.2	2.0–7.5	2.4–7.3	1.9–7.7	7.0–16.6
Percentile	10.0	0.0	0.0	0.0	0.0	0.0	0.0	0.0	10.0	0.0	0.0	0.0	0.0	0.0	0.0	0.0
	25.0	0.0	0.0	0.0	0.0	0.0	0.0	0.0	25.0	0.0	0.0	0.0	0.0	0.0	0.0	0.0
	50.0	0.0	0.0	0.0	0.0	0.0	0.0	0.0	50.0	0.0	0.0	0.0	0.0	0.0	0.0	0.0
	75.0	0.0	0.0	0.0	0.0	0.0	7.0	8.0	75.0	0.0	0.0	0.0	0.0	3.8	0.0	13.0
	90.0	9.4	8.3	10.1	12.0	14.0	38.2	31.0	90.0	2.0	0.0	12.4	12.7	14.7	13.9	49.6

*CI* confidence interval
